# Laryngeal Diphtheria Treated by Antitoxin and Calomel Fumigation*Case reported before the Richmond Academy of Medicine and Surgery, June 8, 1897.

**Published:** 1897-07

**Authors:** E. C. Levy

**Affiliations:** Richmond, Va., Professor of Histology, Pathology, and Bacteriology at the Medical College of Virginia


### LARYNGEAL DIPHTHERIA TREATED BY ANTITOXIN AND CALOMEL FUMIGATION.*

By E. C. LEVY, M.D., Richmond, Va.,

Professor of Histology, Pathology, and Bacteriology at the Medical College of Virginia.

I wish to report this case, not because it presents any remarkable phases to those of you who have availed yourselves of the remedies which were used in its treatment, but merely to illustrate how the advent of these additions to our means of combating this dread disease has revolutionized its treatment. So strong a statement as this would not be justified were it the outcome of the observation of a single case, but I make it confidently because the result in the case I wish to report is in conformity with my previous experience in a large number of cases at the Willard Parker Hospital, New York, and with the reports of thousands of cases treated by others during the past few years.

Gladys E., age 4 years, 6 months, was seen by Dr. Upshur on the morning of Friday, May 28th. The following history was obtained from the parents: Previous history negative. Had been suffering with a slight "cold" for three days. On night of 26th had mild attack of croup. Was much better next day, but complained of throat hurting her. Had another attack of croup on night of Thursday, 27th, which did not improve on following day. From time of first attack of croup child was drowsy and languid. On morning of Friday, the 28th, the parents took her to a neighboring drug-store for advice, but to the credit of this member of a much-abused fraternity, the druggist's only advice was to send for a doctor.

---

*Case reported before the Richmond Academy of Medicine and Surgery, June 8, 1897.

When Dr. Upshur saw the case there was marked obstructive dyspepsia, and a small patch of pseudo-membrane on the left tonsil. These symptoms led the doctor to a diagnosis of the case as one of diphtheria, and he prescribed a mixture of tr. ferri. chlor., hydrarg. chlor. cor., and glycerine. In the afternoon he sent for me to take a culture. By this time the croup symptoms were much more pronounced, and the pseudo-membrane above had spread rapidly, both tonsil and portions of the soft palate being covered with a thick yellow deposit. At 9 p.m. Dr. Upshur telephoned for me to see the case in consultation. I advised the immediate use of antitoxin. As Dr. Upshur was to leave town on the following day he requested me to take charge of the case. I at once ordered the neck poulticed, and injected 1,800 units of antitoxin as soon as it could be procured, continuing the mixture which Dr. Upshur had prescribed. (I injected at the same time 200 units into the two-year-old sister of the patient, who had been into the same room, and whom it was impossible to isolate.) During the early part of the night there was considerable dyspnea, with retraction of the supra-sternal notch and supra-clavicular regions, and I was prepared to intubate should it become necessary. Later on the dyspnea became less, and I left Dr. Dorsey Morris in charge for the night. On seeing the child the following morning (Saturday), Dr. Morris reported that she had passed a comparatively comfortable night, and had coughed up a good sized piece of membrane. During the day she continued to do well. The culture taken on the preceding day showed an abundant growth of Klebs-Lœffler bacilli. At 4:30 p.m. I injected 1,000 units more of antitoxin. At midnight I again saw the child, and found her suffering with urgent dyspnea, which, the nurse reported, had begun to appear about 9 o'clock. I discontinued the iron and bichloride mixture, and ordered a mixture of syrup of epicac and compound syrup of squills, to be given every hour to the point of nausea.

I was sent for hurriedly four hours later, and found the child much worse. Retraction was now marked, the entire sternum sinking in at each inspiration, and there was beginning cyanosis.

The necessity for giving relief was imperative. I had the instruments all prepared for intubation, but decided, before resorting to operative measures, which always increase the gravity of a case, especially in private practice, to try calomel fumigation, believing as I did that there was present a considerable spasmodic element. An improvised tent was accordingly rigged up over the child and twenty grains of calomel sublimated under it. In five minutes, improvement could be noticed; in fifteen minutes, all urgent symptoms had subsided, and in half an hour the child was asleep and breathing so quietly that her respirations could not be heard across the room. On paying my afternoon call, I found the child sitting up in bed, playing with her doll and begging, with quite a loud voice, to be allowed to sit at the table. On the following day, I began the administration of strych. sulph. gr. 1-128 four times a day. The child has made an uninterrupted recovery. The membrane disappeared from the throat on last Wednesday, the 2d, but the bacilli are still persistent. The little sister of the patient, to whom I gave 200 units of antitoxin, has been in the sick-room the entire time, but has developed no symptoms of the disease.

This is the third case of laryngeal diphtheria I have seen in consultation during the past six months. The other two I had the honor of reporting to the Academy on a previous occasion. The first of these cases was not a very severe one, although the dyspnea was very urgent at times, and the child made a prompt recovery under antitoxin, general sustaining treatment, and steam inhalations. The second was a case seen in Manchester in consultation with Dr. Mathews. This case required intubation at once, and died, sixteen hours after I first saw it, from extension of membrane far down into the trachea.

The case I have reported here to-night is a most instructive one, and illustrates forcibly the advantages of antitoxin treatment. Those who oppose this remedy must show us equal results obtained by other methods before any theoretical objections to its use will have weight. It also illustrates the point to which I have directed attention before on the floor of the Academy, viz.: that the use of antitoxin does not justify us in neglecting a single one of the other measures which time and experience have shown to be of benefit. In this case, had the other remedies, especially the calomel fumigation, not been resorted to, I believe the child would not have recovered. On the other hand, had antitoxin injections not been employed, I am confident that all the other measures would have been unavailing. Any plan of treatment which, within forty hours, may convert a serious case of a most serious disease, laryngeal diphtheria, into one in which a favorable prognosis can be given must commend itself to the attention of every thoughtful physician.

---

### TREATMENT OF GONORRHEAL OPHTHALMIA.

Dr. D. T. Vail, of Cincinnati, closes a good paper (*Lancet-Clinic*, April 3) with this summary:

1. The general practitioner should always warn his gonorrhea and leucorrhea patients of the danger of inoculating their eyes.

2. As the family physician is usually the first consulted, he has the golden opportunity which the first hours afford. He should seal the unaffected eye at once.

3. It is well to bear in mind that all cases of purulent ophthalmia are not gonorrheal; on the contrary, only a very small percentage are.

4. For diagnostic and scientific reasons, microscopical examination of the discharge should be made.

5. For the cornea to escape involvement is the great exception.

6. The best early treatment, in my judgment is: (*a*) Leeching; (*b*) continuous iced applications day and night; (*c*) nitrate of silver, 2 to 4 per cent. solution, applied to the everted eyelids once or twice a day; (*d*) non-irritating gentle flushings of the eye every few minutes; (*e*) canthotomy downwards and outwards to liberate the lower lid.

